# ‘Long COVID’: persistent COVID-19 symptoms in survivors managed in Lagos State, Nigeria

**DOI:** 10.1186/s12879-020-05716-x

**Published:** 2021-03-25

**Authors:** Bodunrin Osikomaiya, Olufemi Erinoso, Kikelomo Ololade Wright, Aina Olufemi Odusola, Babafemi Thomas, Oluwatosin Adeyemi, Abimbola Bowale, Olusola Adejumo, Ayodeji Falana, Ismail Abdus-salam, Olusegun Ogboye, Akin Osibogun, Akin Abayomi

**Affiliations:** 1 Lagos Blood Transfusion Services, Lagos, Nigeria; 2 Lagos State University Teaching Hospital, Lagos, Lagos State Nigeria; 3 Health Service Commission, Lagos, Lagos State Nigeria; 4 Mainland Hospital, Yaba, Lagos, Nigeria; 5 Lagos State Ministry of Health, Lagos, Nigeria; 6 College of Medicine, University of Lagos, Lagos, Nigeria

**Keywords:** COVID-19, Survivors, Persistent symptoms

## Abstract

**Background:**

Coronavirus disease once thought to be a respiratory infection is now recognised as a multi-system disease affecting the respiratory, cardiovascular, gastrointestinal, neurological, immune, and hematopoietic systems. An emerging body of evidence suggests the persistence of COVID-19 symptoms of varying patterns among some survivors. This study aimed to describe persistent symptoms in COVID-19 survivors and investigate possible risk factors for these persistent symptoms.

**Methods:**

The study used a retrospective study design. The study population comprised of discharged COVID-19 patients. Demographic information, days since discharge, comorbidities, and persistent COVID-19 like symptoms were assessed in patients attending the COVID-19 outpatient clinic in Lagos State. Statistical analysis was done using STATA 15.0 software (StataCorp Texas) with significance placed at *p*-value < 0.05.

**Results:**

A total of 274 patients were enrolled in the study. A majority were within the age group > 35 to ≤49 years (38.3%), and male (66.1%). More than one-third (40.9%) had persistent COVID-19 symptoms after discharge, and 19.7% had more than three persistent COVID-like symptoms. The most persistent COVID-like symptoms experienced were easy fatigability (12.8%), headaches (12.8%), and chest pain (9.8%). Symptomatic COVID-19 disease with moderate severity compared to mild severity was a predictor of persistent COVID-like symptoms after discharge (*p* < 0.05).

**Conclusion:**

Findings from this study suggests that patients who recovered from COVID-19 disease may still experience COVID-19 like symptoms, particularly fatigue and headaches. Therefore, careful monitoring should be in place after discharge to help mitigate the effects of these symptoms and improve the quality of life of COVID-19 survivors.

## Background

COVID-19 is currently spreading rapidly with increasing incidence across most countries of the world [1]. Nigeria, the most populous African country, has recorded 42,208 confirmed cases since the index case in February 2020, and 19,004 recovered cases from the infection as of July 30th 2020 [2]. Lagos, the most populous State in Nigeria, and the epicentre of coronavirus disease 2019 (COVID-19) in Nigeria has recorded 14,954 cases and 192 deaths [2]. However, these figures could be much higher as they are subject to challenges of limited testing capacities which many countries, including Nigeria, currently face amidst potential asymptomatic infections.

The spectrum of clinical presentation of COVID-19 ranges from the asymptomatic, to symptomatic with varying levels of severity depending on age, comorbid conditions, and basal metabolic index [3, 4]. If symptomatic, COVID-19 patients may present with fever, dry cough, anosmia, fatigue as common symptoms, amongst others. Less common symptoms include aches and pains, sore throat, nausea, vomiting and diarrhoea, conjunctivitis, headache, and a skin rash or discolouration of fingers or toes [3, 5–7]. Associated shortness of breath and difficulty in breathing indicate severe disease [3, 4]. While most infected people develop mild to moderate illness and recover without requiring hospitalisation, others become sick enough to be hospitalised. Few succumb to the disease as a result of severe irreversible respiratory compromise, the lungs being the most severely affected body organ [8]. Emerging pathologic evidence suggests increased coagulability of many victims’ blood as autopsy findings reveal blood clots in the lungs [9].

Typically, COVID-19 symptoms manifest in individuals for 14 days from onset of symptoms, after which, symptoms expectedly subside or resolve completely, and infectiousness abates [8, 10]. An emerging body of evidence, however, suggests the persistence of post-infection clinical symptoms of varying consistencies and durations among some survivors [11–16].

In this study, we describe persistent symptoms in patients who had recovered from COVID-19. Our objectives were to highlight associations between socio-demographic characteristics and comorbidities with persistent symptoms in COVID-19 survivors. Findings from our study may provide additional evidence to guide public health measures and policies, as well as build on the current knowledge of the long-term effects of COVID-19.

## Methods

### Study design

A retrospective study using de-identified data of cases from the Lagos State COVID-19 outpatient clinic seen between April and June 2020.

### Study setting

On April 27th 2020, the Lagos State COVID-19 incident command established a COVID-19 outpatient clinic for all COVID-19 survivors discharged from the six isolation facilities being run in the State. Patients were referred to the Outpatient clinic two weeks after discharge from COVID-19 isolation facilities. Discharge from the isolation facilities between March and May 2020 was based on the World Health Organization (WHO) criteria: Three days after resolution of symptoms and/or Two negative RT-PCR SARS-CoV-2 results, at least 24 h apart. The term ‘*survivor*’ (as used throughout the paper) is used to describe patients who met the above criteria before discharge from isolation and treatment facilities in Lagos State, Nigeria.

### Data collection

A thorough clinical history and physical assessment was conducted for all patients at the outpatient clinic. Information was obtained on the presence or absence of COVID-19 symptoms, general symptoms, and the nature of symptoms if any. This information was reconciled with prior demographic information and medical history for each patient and entered in the outpatient database. The term ‘*Persistent symptoms*’ (as used throughout the paper) was used to describe patients’ self-reported COVID-19 like symptoms reported during clinical review and physical assessment at the Post COVID-19 clinic. Information on the presence or absence of persistent symptoms before COVID-19 infection was confirmed before the diagnosis of persistent COVID-19 symptoms to rule out potential confounding comorbidities or concurrent infections. Participants were also grouped based on the severity at the time of initial COVID-19 diagnosis using the WHO criteria [17]: Mild- Symptomatic patients with no evidence of viral pneumonia or hypoxia; Moderate – SpO2 > 90%; fever, cough, dyspnea, fast breathing (clinical signs of pneumonia) but no signs of severe pneumonia; Severe - SpO2 < 90%; Adolescent or adult with clinical signs of pneumonia (fever, cough, dyspnea, fast breathing); severe respiratory distress; Asymptomatic- No symptoms.

### Statistical analysis

Demographic information, medical history and persistence of symptoms were analysed using descriptive statistics. Univariable and multivariable logistic regression models were used to investigate the association between demographic information, comorbidities and the presence or absence of persistent COVID-19 symptoms. *P* values of < 0.05 were considered significant, and tests were 2- tailed. Statistical analysis was done using STATA 15.0 software (StataCorp LLC Lakeway Drive, College Station, Texas).

### Ethics

Ethical approval was obtained from the Lagos State University Teaching Hospital Health Research Ethics Committee. Approval number: LREC/06/10/1345.

## Results

A total number of 274 COVID-19 survivors were assessed in this study. The mean age of respondents was 41.8 years (SD ± 11.8) with a range of 10 to 83 years. Majority of respondents were male (66.1%) and aged between 36 and 49 years (38.3%). The median number of days after discharge with COVID-19 like symptoms was 15 days (IQR 14–17 days) (Table 1). Among the study participants, a majority (50.7%) had a mild form of the disease at the initial time of COVID-19 diagnosis (Table 1).

**Table 1:** Clinical characteristic of cases

Patient Characteristics	N = 274 (%)
**Age** [in years]	
≤ 35	92 (33.6)
> 35–49	105 (38.3)
50 and above	77 (28.1)
**Sex**	
Male	181 (66.1)
Female	93 (33.9)
**Comorbidity**	
Present	59 (21.5)
Absent	215 (78.5)
^a^**Comorbidity**	*n* = 59
Diabetes (DM)	9 (15.3)
Hypertension (HTN)	43 (72.9)
Asthma	2 (3.4)
Peptic Ulcer Disease	4 (6.8)
Rheumatoid Arthritis	3 (5.1)
^b^Other	5 (8.5)
**Initial COVID-19 severity**	
Asymptomatic	21 (7.7)
Mild	139 (50.7)
Moderate	107 (39.0)
Severe	7 (2.6)
**Number of comorbid conditions**	
No comorbid condition	215 (78.5)
1 comorbid condition	52 (19.0)
2 or more comorbid conditions	7 (2.5)
**Persistent symptoms**	
Present	112 (40.9)
Absent	162 (59.1)
**Number of Persistent symptoms**	
Absent	162 (59.1)
1 persistent symptom	31 (11.3)
2 persistent symptoms	27 (9.9)
3 or more persistent symptoms	54 (19.7)
Days after discharge, median [mean; SD]	15 days [mean: 15.6; SD ± 6.6]

^a^ Three participants had both DM and HTN. ^b^ Other: One patient each had sickle cell disease, Human Immunodeficiency Virus, chronic heart disease, glaucoma, and a psychiatric condition

### Clinical characteristics

More than two-thirds (78.5%) of the study population had no underlying comorbid condition (Table 1). Among respondents with underlying comorbidities (*n* = 52), the most common comorbidities were hypertension (72.9%) and diabetes (15.3%), respectively (Table 1). Over half (59.1%) of the study population had no persistent COVID-19 symptoms. Among respondents with persistent symptoms (*n* = 112), a majority (48.2%) had three or more persistent COVID-like symptoms (Table 1). Of all cases reviewed (*n* = 274), the most common symptoms were easy fatigability (12.8%), headache (12.8%), chest pain (9.8%), and insomnia (9.8%) (Table 2).

**Table 2:** Self-reported symptoms of COVID-19 Survivors

System	Symptom	N = 274 (%)
General	Fever	17 (6.2)
	Easy fatigability	35 (12.8)
	Weight loss	17 (6.2)
	Malaise	15 (5.5)
	Myalgia	24 (8.8)
	Excessive night sweat	10 (3.7)
Respiratory	Cough	25 (9.2)
	Dyspnea	26 (9.5)
	Chest pain	27 (9.8)
Otolaryngology (ORL)	Sore throat	11 (4.0)
	Dysgeusia	7 (2.6)
	Anosmia	5 (1.8)
Gastrointestinal	Loss of appetite	24 (8.8)
	Vomiting	2 (0.73)
	Nausea	6 (2.2)
	Diarrhoea	11 (4.0)
	Abdominal discomfort	17 (6.2)
Cardiovascular	Palpitations	20 (7.4)
Neurologic	Headache	35 (12.8)
	Insomnia	27 (9.8)
	Attention or memory deficit	14 (5.2)
	Dizziness	8 (2.9)
	Visual blurring	6 (2.2)
	Tremulousness	2 (0.7)
	Vertigo	3 (1.1)

### Association between the presence of persistent symptoms and demographic and medical history among symptomatic COVID-19 cases

Table 3 demonstrates there was no association between age, sex, a history of hypertension, diabetes or multiple comorbidities with the presence of persistent COVID-19 like symptoms among symptomatic COVID-19 cases (*n* = 253) on univariable logistic regression models. However, participants with moderate disease severity at the time of initial COVID-19 diagnosis had increased odds of persistent COVID-19 symptoms compared to the participants with mild disease severity (OR: 2.17; 95 CI: 1.30, 3.64; p: 0.003). After adjusting for age, sex, number and type of comorbidities (hypertension and diabetes), moderate disease severity at the time of initial COVID-19 diagnosis remained significantly associated with higher odds of persistent COVID-19 like symptoms (aOR: 2.03; 95% CI: 1.19, 3.47; p: 0.009) (Table 3).

**Table 3:** Logistic regression model demonstrating the odds of Persistent COVID-19 symptoms after discharge

Variables	OR (95% CI)	***p***-value	aOR (95% CI)	***p***-value
**Age**	1.01 (0.99, 1.03)	0.30	1.01 (0.99, 1.04)	0.38
**Sex**				
Female	1 [reference]		1 [reference]	
Male	0.63 (0.37, 1.07)	0.09	0.64 (0.37, 1.11)	0.11
**Comorbidity**				
No comorbidities	1 [reference]		1 [reference]	
1 comorbidity	0.78 (0.41, 1.50)	0.46	0.36 (0.07, 1.81)	0.22
2 comorbidities	7.57 (0.89, 64.03)	0.06	1.73 (0.08, 35.31)	0.72
No history of Hypertension	1 [reference]		1 [reference]	
History of Hypertension	1.17 (0.59, 2.30)	0.65	1.89 (0.33, 10.84)	0.47
No history of Diabetes Mellitus	1 [reference]		1 [reference]	
History of Diabetes Mellitus	3.93 (0.78, 19.88)	0.10	3.72 (0.38, 36.63)	0.26
^a^**Initial severity**				
Mild	1 [reference]		1 [reference]	
Moderate	2.17 (1.30, 3.64)	0.003*	2.03 (1.19, 3.47)	0.009*
Severe	4.59 (0.86, 24.55)	0.08	3.77 (0.66, 21.35)	0.13

*p*-value < 0.05*. OR odds ratio*, aOR* adjusted odds ratio*, 95% CI* 95% confidence interval*,* Model adjusted for age, sex, and presence of comorbid conditions. ^a^ Initial severity*:* Mild moderate and severe COVID-19 at time of initial diagnosis, excluding asymptomatic cases *(n* = 21*).* Multivariable logistic regression Model has *p-*value- < 0.009 and Pseudo R^2^ of 5.9%

## Discussion

This study assessed the pattern of COVID-19 like symptoms and their association with demographic factors and comorbidities in a cohort of 274 survivors followed up at a COVID-19 outpatient clinic in Lagos State, Nigeria. About four out of ten survivors remained symptomatic for atleast two weeks after discharge, with the majority of them presenting with more than three symptoms. Easy fatigability, headache, chest pain and insomnia were the most common symptoms. Further, there was a significant association between severity of COVID-19 disease at initial diagnosis with the presence of persistent COVID-19 like symptoms after hospitalisation. Our findings align with reports on COVID-19 symptoms in survivors based on recent studies from the United States, the United Kingdom, and Italy [11, 12, 14, 15].

The most common symptom manifested by survivors in our study was easy fatigability. Carfi et al. [11] also reported easy fatigability in a review of COVID-19 survivors, likewise, Tenforde et al. in a review of cases in the United States [12]. Also, a COVID-symptom study app in the United Kingdom which tracks patients for weeks and months has reported similar symptoms, as in this study [13]. From other peer-reviewed studies and this study, easy fatigability was a consistently identified symptom in COVID-19 survivors [11–15]. Further, several studies have identified chronic pain and psychological distress (anxiety, depression, and post-traumatic stress) in COVID-19 survivors [18–21]. This underscores the importance of channelling resources to the long-term follow-up and care of COVID-19 survivors to support their return to a normal state of health. Recently the quality of life and rehabilitation of COVID-19 survivors after discharge has generated research interests [11, 22–25]. Our study identified neurologic symptoms in 39.1% of the COVID-19 survivors reviewed, symptoms such as headaches, insomnia, and attention deficits. These symptoms can potentially preclude a safe and effective return of survivors to usual life. More studies are needed to assess predictors of persistent symptoms, as well as the quality of life in COVID-19 survivors in Nigeria and globally.

Based on our findings, there was no significant association between demographic factors and comorbidities such as hypertension or diabetes and the presence of persistent symptoms in COVID-19 survivors. However, a prior study reported the presence of more than three underlying medical conditions as significant factors in predicting a delayed return to the normal state of health 14–21 days after testing [12], which is contrary to our study where two or more comorbidities was not significantly associated with persistent COVID-19 like symptoms. Further, participants that were symptomatic, with moderately severe forms of COVID-19 disease, at the time of initial diagnosis had higher odds of persistent COVID-19 like symptoms after discharge compared to participants that were mildly symptomatic at the time of initial diagnosis. The relationship between COVID-19 severity and increased odds of persistent symptoms may be explained by the immune response to the SARS-CoV-2 virus which stimulates the production of cytokines and other inflammatory mediators, with higher concentrations found in those with a more severe clinical form [3, 26–28]. The multi-systemic inflammatory response to the virus may also be responsible for persistent COVID-19 symptoms in survivors [28–31].

Persistent COVID-19 symptoms- ‘long COVID’- have been reported to have similarities with other syndromes such as myalgic encephalomyelitis, or chronic fatigue syndrome which occur after viral infections [3, 11, 14] However, the exact pathophysiology of ‘long COVID’ is not yet understood. Probable mechanisms described in the literature include vascular inflammation, detected using 2-deoxy-2-[18F] Fluoro-D-glucose [30], hyper-inflammation from cytokines storm which alters the immune response function, or endothelial dysfunction caused by the action of the SARS-CoV-2 on ACE2 receptors [30, 31]. These mechanisms all support the evidence that COVID-19 is primarily a multi-systemic disease, with varied manifestations [32]. Further, to better understand the entity ‘long COVID’, it is important to distinguish between symptoms from persistent chronic inflammation, and symptoms which may arise as sequelae of organ damage (pulmonary fibrosis and subsequently cough), and other persistent symptoms from factors such as hospital-stay and social isolation (nutritional anaemia, weight loss, muscle wasting) [16].

Of interest is the potential effect of the public health messaging on rapid recovery from COVID-19. While a majority of COVID survivors with mild cases may recover rapidly in about two weeks [26], our evidence suggests that a significant number experience long-term symptoms. The unintended effect of messaging on rapid recovery can be psychosocial, as survivors could begin to worry about their normalcy when experiencing delayed physical or mental recovery. Therefore, effective strategies are needed to accurately coin messaging on COVID-19 recovery and educate survivors to ease the return to their usual way of life.

The current study has limitations. For example, the study depended on self-reported persistent COVID-19 symptoms. However, the diagnosis was carefully made to avoid potential confounders such as symptoms which had been present before SARS-COV-2 infection, concurrent infection from other viral agents, and underlying comorbidities. In addition, the sample population was relatively small, and the retrospective design, as well as the convenient sampling method, may not be generalisable to the over 40,000 confirmed cases of COVID-19 in the country [2]. Further, some self-reported symptoms might be associated with standard therapeutic agents administered to the patients during isolation or treatment in Lagos State [33]. For example, diarrhoea associated with lopinavir/ ritonavir, which was administered as the standard of care between April and May 2020 in Lagos State. However, this association is unlikely because patients presented at the COVID-19 follow-up clinic 14 days or more after therapy and discharge, and effects would have subsided.

## Conclusion

In conclusion, this study presents early evidence to support the emerging literature on the persistent manifestation of clinical symptoms among survivors of COVID-19 disease in Lagos State, Nigeria. In the cases reviewed, most survivors with persistent symptoms had more than two self-reported symptoms. Easy fatigability, headaches, chest pain and insomnia, were the most common symptoms. As interest and calls for a review of the current evidence of symptoms in survivors emerge [13, 34], further research is needed to identify factors associated with persistent COVID-19 like symptoms in survivors- termed ‘long COVID’ [34]. Evidence from these studies can guide policies and interventions aimed at improving the quality of life of survivors and return to usual health.

## Data Availability

The datasets during and/or analysed during the current study available from the corresponding author on reasonable request.

## Abbreviations

ACE2: Angiotensin-Converting Enzyme- 2
aOR: Adjusted odds ratio
COVID-19: Coronavirus disease 2019
DM: Diabetes Mellitus
HTN: Hypertension
OR: Odds ratio
SARS-CoV-2: Severe acute respiratory syndrome coronavirus 2
SpO2: Oxygen saturation
WHO: World Health Organization
